# Differential responses to thrombospondin-1 and PDGF-BB in smooth muscle cells from atherosclerotic coronary arteries and internal thoracic arteries

**DOI:** 10.1038/s41598-024-66860-x

**Published:** 2024-07-09

**Authors:** Alokkumar S. Pathak, George A. Stouffer

**Affiliations:** Division of Cardiology and McAllister Heart Institute, University of North Carolina, Chapel Hill, NC 27599-7075 USA

**Keywords:** Smooth muscle, Thrombospondin, Internal thoracic, Atherosclerosis, PDGF-BB, Cell growth, Mechanisms of disease, Cardiology

## Abstract

Atherosclerosis is rare in internal thoracic arteries (ITA) even in patients with severe atherosclerotic coronary artery (ACA) disease. To explore cellular differences, ITA SMC from 3 distinct donors and ACA SMC from 3 distinct donors were grown to sub-confluence and growth arrested for 48 h. Proliferation and thrombospondin-1 (TSP1) production were determined using standard techniques. ITA SMC were larger, grew more slowly and survived more passages than ACA SMC. ACA SMC had a more pronounced proliferative response to 10% serum than ITA SMC. Both ACA SMC and ITA SMC proliferated in response to exogenous TSP1 (12.5 µg/ml and 25 µg/ml) and platelet derived growth factor-BB (PDGF-BB; 20 ng/ml) but TSP1- and PDGF-BB-induced proliferation were partially inhibited by anti-TSP1 antibody A4.1, microRNA-21(miR-21)-3p inhibitors and miR-21-5p inhibitors in each of the 3 ACA SMC lines, but not in any of the ITA SMC lines. PDGF-BB stimulated TSP1 production in ACA SMC but not in ITA SMC but there was no increase in TSP1 levels in conditioned media in either SMC type. In summary, there are significant differences in morphology, proliferative capacity and in responses to TSP1 and PDGF-BB in SMC derived from ITA compared to SMC derived from ACA.

## Introduction

The internal thoracic artery (ITA) is resistant to the development of atherosclerosis compared to other arteries in the body including coronary arteries, carotid arteries and lower extremity arteries. Prevalence rates of histologically proven atherosclerotic lesions in ITA have ranged between 3.1 and 4.2% in unselected individuals^1,2^ and 0.7% and 7% in patients with multivessel coronary artery disease^3,4^. Differences in arterial predisposition to atherosclerosis begins at an early age; in a study comparing intimal thickening of the left anterior descending (LAD) coronary artery and the ITA in 352 patients of all ages, the LAD showed early development of intimal thickening that progressed in severity throughout life, whereas the ITA showed no more than slight changes at any age^5^. The resistance to atherosclerosis in ITA remains largely unexplained as traditional clinical risk factors for atherosclerotic disease (e.g., diabetes, hypertension, tobacco use, hyperlipidemia, family history of premature atherosclerosis) are all systemic implying that there are important differences in responses of specific arteries to athero-stimuli.

Atherosclerosis is a chronic inflammatory disease and smooth muscle cells (SMC) play a major role in all phases of atherogenesis. According to the response to injury hypotheses, contractile SMC are recruited from the media and undergo phenotypic conversion to proliferative synthetic cells as they migrate into the intima^6,7^. Platelet-derived growth factor-BB (PDGF-BB) is a multifunctional growth factor that is upregulated at sites of vascular injury^8–10^ and that stimulates SMC proliferation, migration and dedifferentiation. PDGF-BB elicits a change in SMC phenotype from a differentiated, contractile state to a dedifferentiated, synthetic state through effects mediated by multiple pathways which repress SMC marker gene expression^11^.

Studies comparing rings of freshly isolated saphenous vein and ITA suggested that responses to PDGF-BB differ between SMC from these two sources. Intimal hyperplasia was amplified in saphenous vein rings following the addition of PDGF–BB whereas responses of ITA rings were much less pronounced, even at much higher doses of PDGF-BB^12^. Studies comparing SMC cultured from ITA or saphenous vein reported that proliferative responses to PDGF-BB were two fold higher in SMC isolated from saphenous vein than SMC isolated from ITA from the same donor^13^ and a robust proliferative response to PDGF-BB by saphenous vein SMC but no proliferative response to PDGF-BB by ITA SMC^14^.

The majority of studies of SMC from ITA have compared them to SMC from saphenous veins and there are relatively few studies comparing SMC from ITA to SMC from coronary arteries. Given the marked difference in propensity to atherosclerosis of coronary arteries and ITA, and the major role that PDGF-BB plays in atherogenesis, the present studies compared responses to PDGF-BB of SMC isolated from ACA to SMC isolated from ITA.

## Materials and methods

### Reagents

Reagents were obtained from the following sources: thrombospondin-1 (TSP1; Athens Research & Technology, Athens, GA), platelet-derived growth factor-BB (PDGF-BB; R&D Systems, Minneapolis, MN), anti-TSP1 mouse anti-human monoclonal antibodies A4.1 (Invitrogen, Waltham, MA), and monoclonal anti-beta-actin antibody (Sigma Aldrich, St. Louis, MO). Recombinant monoclonal anti-transferrin antibody was obtained from Abcam (Boston, MA), a rabbit monoclonal anti-myosin heavy chain antibody was obtained from Invitrogen (catalog number 702544; Waltham, MA) and rabbit monoclonal anti-Sox-10 antibody (catalog number 89356) and rabbit monoclonal anti-S100B antibody (catalog number 9550) were obtained from Cell Signaling Technology (Danvers, MA). Mouse monoclonal antibodies to TSP1, calponin, α-actin, p16^INK4A^, and p21^Cip1^ were obtained from Santa Cruz Biotechnology, Dallas, TX. Mouse IgG kappa binding protein as anti-mouse secondary and anti-rabbit secondary antibody were obtained from Santa Cruz Biotechnology, Dallas, TX. Complete blots are provided in the Supplementary Figure.

### Cell culture

Cell lines of human ACA SMC from 3 distinct donors (Cat# 350q-05a) and cell lines of ITA SMC from 3 distinct donors (Cat# 358-05a) were obtained from Cell Applications (San Diego, CA). The ACA SMC were obtained from a 53 years old male with hypertension, tobacco use (3–4 packs per day for 35 years) and extensive coronary artery disease (ACA SMC line 1), a 56 years old female with tobacco use (0.5 packs per day for 18 years) who died of an intracerebral hemorrhage (ACA SMC line 2) and a 38 years old male with hypertension and tobacco use (2–3 packs per day × 17 years) who died of an intracerebral hemorrhage (ACA SMC line 3). The ITA SMC were obtained from a 34 years old female with tobacco use (1 pack per day for an unclear duration of time) and lung cancer who died after a cardiac arrest (ITA SMC line 1), a 50 years old male without any risk factors for ACA disease who died of a gunshot wound (ITA SMC line 2) and a 35 years old male without any risk factors for ACA disease who died in a motor vehicle accident (ITA SMC line 3). Schwann cells and HeLa cells were obtained from American Type Culture Collection (Manassas, VA). For proliferation assays, SMC were plated at equal seeding density, grown to subconfluence, and growth arrested for 48 h in 0.5% FBS quiescent medium prior to treatment. Cell numbers were counted using a TC20 Bio-Rad cell counter. Western blotting and conditioned media experiments were performed as previously described^15,16^.

### Quantitative real-time PCR (qRT-PCR)

Total RNA was extracted from SMC by use of RNeasy Mini Kit (Qiagen, Hilden Germany) and quantified using a NanoDrop spectrophotometer (ThermoFisher Scientific, Waltham MA). Quantitative real-time PCR was performed as previously described^17^. Briefly, for detection of the primary miR-21 (pri-miR-21) and pre-miR-21, 1 µg total RNA was reverse transcribed to cDNA by use of universal cDNA synthesis kit (Applied Biosystems, Waltham MA) and real-time PCR was executed with iTaq Universal SYBR Green Supermix (BioRad, Hercules CA) on a Roche 480 Light Cycler. The primer sequences (Sigma-Aldrich) were for pri-miR-21: forward, 5′-TTTTGTTTTGCTTGGGAGGA-3′ and reverse, 5′-AGCAGACAGTCAGGCAGGAT-3′; for pre-miR-21: forward, 5′-TGTCGGGTAGCTTATCAGAC-3′ and reverse, 5′-TGTCAGACAGCCCATCGACT-3′; and for β-actin: forward, 5′-CGTGCGTGACATTAAGGAGA-3′ and reverse, 5′- CACCTTCACCGTTCCAGTTT-3′. β-actin was an internal reference for pri-miR-21 and pre-miR-21 levels. Quantification cycle crossing point (C_p_) values were recorded.

### Transfection with anti-miR21 inhibitors

ACA SMC or ITA SMC cells grown to 70% confluence were transfected with 50 nM anti-*miR-21* miRIDIAN hairpin inhibitors (miR21-3p inhibitor, miR21-5p inhibitor or microRNA hairpin inhibitor negative control; Horizon Discovery, Lafayette, CO), using Lipofectamine RNAiMAX reagent (Invitrogen, Carlsbad, CA), according to manufacturer guidelines. Briefly, Lipofectamine RNAiMAX reagent (3 µl) and anti-miR-21 miRIDIAN hairpin inhibitors stock (1 µl) were diluted in minimum serum medium without antibiotics. Diluted anti-miR-21 miRIDIAN Hairpin Inhibitors or control Si-RNA were added to equal volume (50 µl each) of diluted Lipofectamine RNAiMAX reagent to form a SiRNA-lipid complex and incubated for 5 min at room temperature. SiRNA-lipid complexes were added to cells (50 µl each well to 450 µl culture medium) and incubated for 24 h followed by addition of PDGF-BB (20 ng/ml). After 72 h cells were counted utilizing a TC20 Bio-Rad cell counter.

### Data analysis and statistics

Normally distributed data are presented as mean ± STD and non-normally distributed data as median [25%, 75%]. Comparisons of equal groups was done using Analysis of Variance (ANOVA) followed by Dunnett’s multiple range test. Comparison of unequal groups was done via ANOVA followed by the Tukey multiple range test. ANOVA using Ranks was used for non-Normally distributed continuous variables followed by Holm-Sidak method or Dunn’s Multiple Range test if the groups were unequal in size. Mantel–Haenszel Chi-Square was used to analyze differences between categorical variables. Differences were considered significant at a *p*-value of ≤ 0.05.

## Results

### Morphology and proliferative responses to 10% serum were different in ACA SMC and ITA SMC

**Figure 1:**
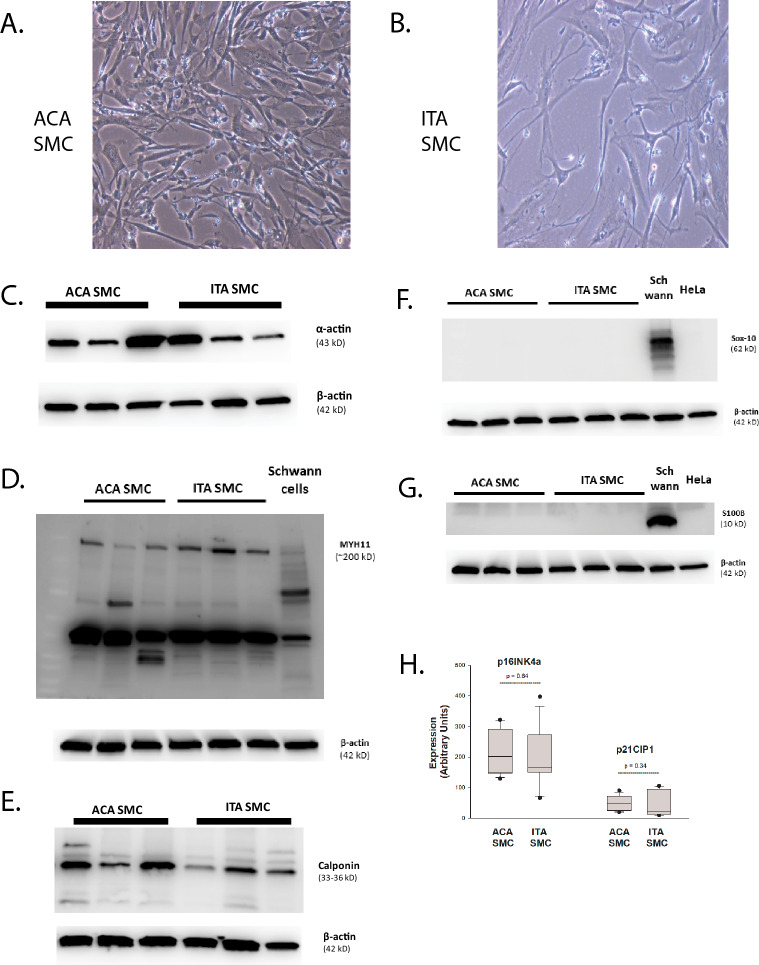
Morphology and SMC markers in human ITA SMC and ACA SMC. Human SMC derived from 3 distinct ACA donors and 3 distinct ITA donors were plated in T75 flasks with growth medium. Schwann cells and HeLa cells were used as controls. Representative photomicrographs of ACA SMC (**A**) and ITA SMC (**B**) taken at subconfluence are shown. Expression of α-actin (**C**), myh-11 (**D**), calponin (**E**), Sox-10 (**F**) and S100B (**G**) were determined as described in Methods and a representative blot from 3 independent experiments for each protein is shown. Expression of the senescence biomarkers p16INK4A and p21Cip1 were determined by Western blotting as described in Methods for growth arrested ACA SMC and ITA SMC. Expression was quantified and normalized to β-actin expression. Results from 3 independent experiments are shown in H.

There were significant differences in morphology when ITA SMC and ACA SMC were grown to subconfluence. ACA SMC isolated from 3 different donors had a classic SMC “hill-and-valley” pattern (Fig. 1A) whereas ITA SMC isolated from 3 different donors grew as a monolayer and had a more spindle appearance (Fig. 1B). All of the cell lines expressed SMC markers including α-actin (Fig. 1C), myosin heavy chain MHY11 (Fig. 1D) and calponin (Fig. 1E) but not stem cell markers Sox-10 (Fig. 1F) or S100B (Fig. 1G). There was no difference in levels of p16^INK4A^ and p21^Cip1^, biomarkers of senescence, in a pooled analysis of ACA SMC and ITA SMC (Fig. 1H)^18^.

**Figure 2:**
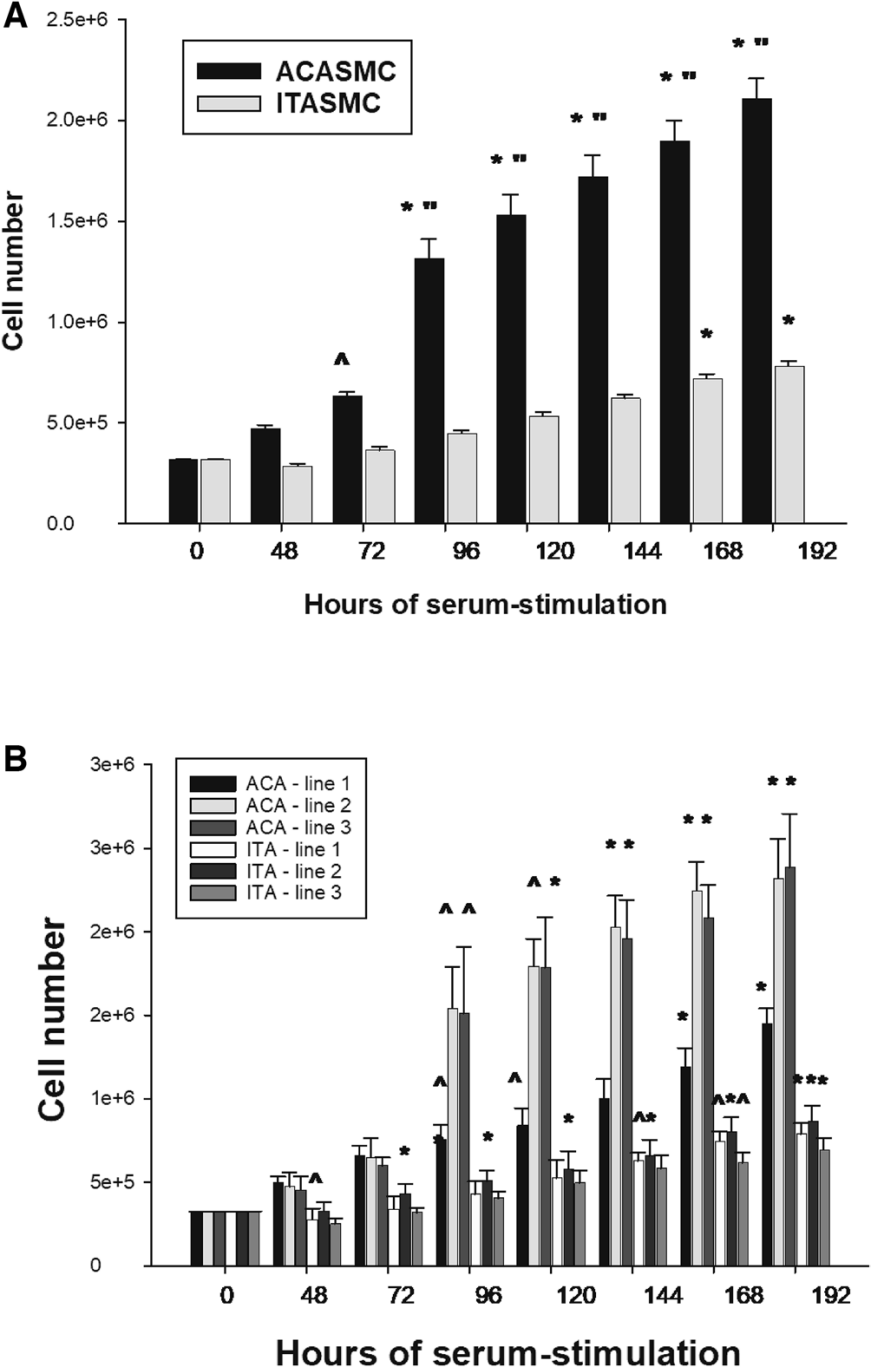
Proliferative responses of human ITA SMC and ACA SMC to FBS. Human SMC derived from 3 distinct ACA donors and 3 distinct ITA donors were plated at equal seeding density in T25 flasks, grown to subconfluence, growth arrested and then exposed to 10% FBS. Cell counts were performed at 48 h, 72 h, 96 h, 120 h, 144 h, 168 h and 192 h. Results of combining data from the 3 ACA SMC lines and the 3 ITA SMC lines are shown in (**A**) and results from 6 to 10 independent experiments with each cell line are shown in (**B**). [SEM is presented in (**A**) and STD is presented in (**B**); ^—*p* < 0.05 vs control; *—*p* < 0.001 vs control; “—*p* < 0.05 vs ITA SMC].

When ACA SMC and ITA SMC were plated at equal densities in T25 flasks and exposed to 10% fetal bovine serum (FBS), ACA SMC had a more pronounced proliferative response than ITA SMC at 72 h, 96 h, 120 h, 144 h, 168 h and 192 h (Fig. 2A). When each ACA and ITA cell line was analyzed separately, proliferative responses to 10% FBS were consistently more robust in the ACA SMC lines (Fig. 2B). Both ACA SMC and ITA SMC proliferated in response to 20 ng/ml of recombinant PDGF-BB but, in contrast to results observed with FBS stimulation, the proliferative responses to PDGF-BB were the same in both ACA SMC and ITA SMC (ACA SMC: 172.1% ± 5.9% vs 162.4% ± 5.5%; *p* = 0.15).

### PDGF-BB-induced proliferation was partially inhibited by anti-TSP1 antibodies in ACA SMC but not ITA SMC

The thrombospondin protein family consists of 5 members, the best studied of which is TSP1 which has been implicated in migration and proliferation of SMC. Several lines of evidence have identified TSP1 as playing an important role in mediating vascular healing in vivo: (a) TSP1 levels are increased at sites of vascular injury including atherosclerotic lesions^19^, occluded SVG^20^, and following balloon injury^16,21,22^ whereas TSP1 levels are negligible in the normal vasculature; and (b) neointimal formation following arterial injury is reduced by pharmacological inhibition of TSP1^23,24^ and in TSP1 knockout mice^24^. Differential effects of autocrine TSP1 in mediating PDGF-BB-induced proliferation of SMC have been observed in prior studies^15,25–27^.

**Figure 3:**
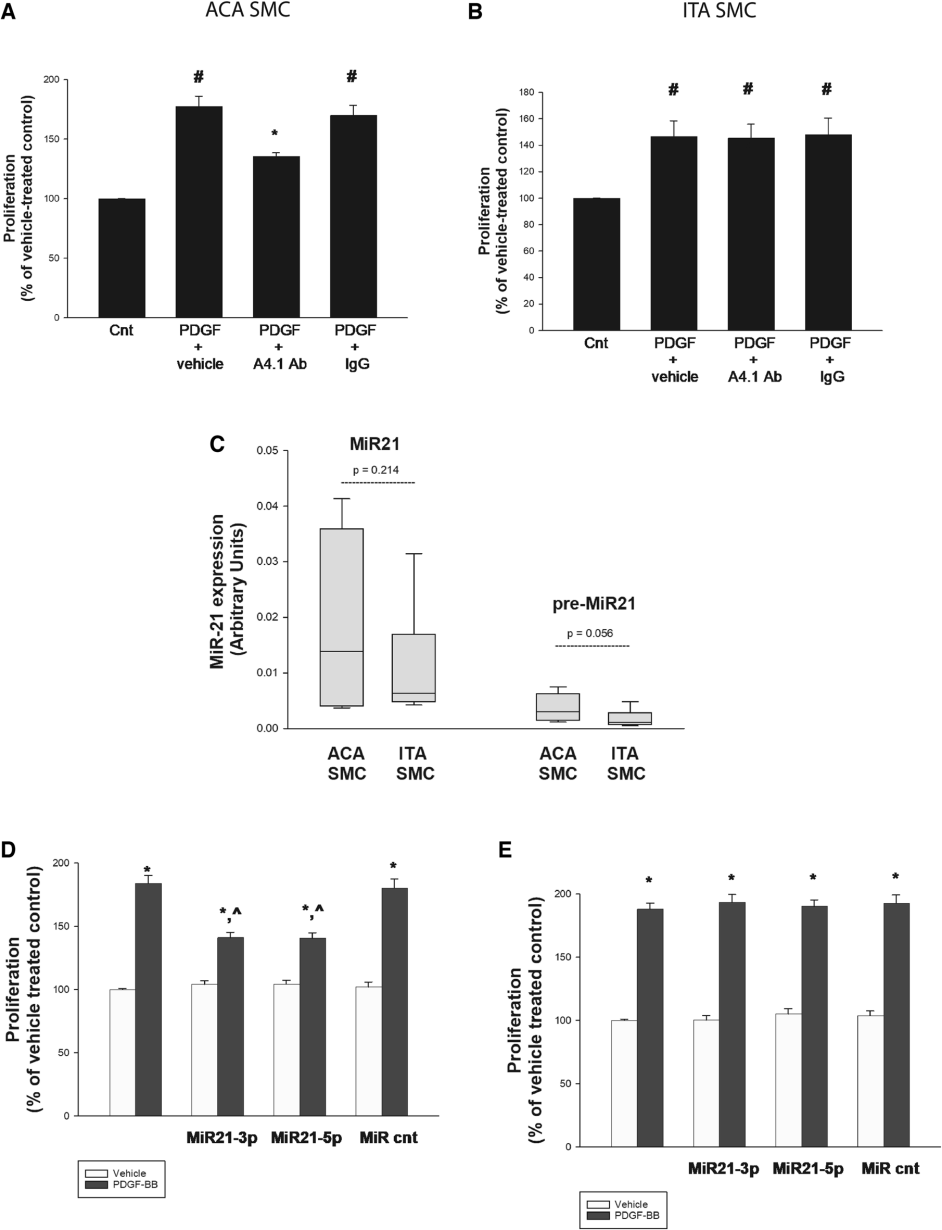
PDGF-BB induced proliferation is inhibited by anti-TSP1 neutralizing antibody A4.1 and by MiR21-3p inhibitors and MiR21-5p inhibitors in ACA SMC but not in ITA SMC. Human SMC derived from 3 distinct ACA donors (**A**, **C** and **D**) and 3 distinct ITA donors (**B**, **C** and **E**) were growth arrested as described in Methods. (**A**) and (**B**): The indicated groups were pretreated with vehicle, anti-TSP-1 antibody A4.1 (10 µg/ml), or a non-specific IgG (10 µg/ml) for 30 min prior to treatment with vehicle or PDGF-BB (20 ng/ml). Cell counts were determined 96 h later. [n ≥ 8 independent experiments; #—*p* < 0.05 compared to Cnt; *—*p* < 0.05 compared to PDGF-BB + IgG]. (**C**): Total RNA was extracted and quantitative RT-PCR performed in triplicate as described in Methods. (**D**) and (**E**): The indicated groups were pretreated with vehicle, 50 nM miR21-3p inhibitor, 50 nM miR21-5p inhibitor or 50 nM MiRCnt for 24 h prior to treatment with vehicle or PDGF-BB (20 ng/ml). Cell counts were determined 96 h later. [n ≥ 8 independent experiments; *—*p* < 0.05 compared to cnt; ^—*p* < 0.05 compared to TSP + MiR cnt].

To explore the role of autocrine TSP1 in PDGF-BB-induced proliferation of ACA SMC and ITA SMC, A4.1, an anti-TSP1 monoclonal neutralizing antibody that recognizes an epitope in the N-terminal half of the central stalk-like region of TSP1 was utilized. A4.1 has been shown to block TSP1-induced proliferation^25,26,28^ and TSP1-induced activity of cyclin-dependent kinase 2 and S-phase entry in rat aortic SMC (RASMC) but effects in human SMC have not been studied^23^. Pre-treatment with A4.1 inhibited PDGF-BB-induced proliferation by 50 ± 5% in ACA SMC but had no effect on PDGF-BB-induced proliferation of ITA SMC. Results were consistent in all 3 ACA SMC lines and in all 3 ITA SMC lines and aggregate data is shown in Fig. 3A and B.

The inhibitory effects of A4.1 on PDGF-BB-induced proliferation of ACA SMC are unlikely to be due to an increase in nonspecific cell detachment or apoptosis as A4.1 at concentrations ten-fold higher (100 µg/ml) than used in the current study did not cause cell detachment visible by light microscopy or detectable by cell adhesion assays in RASMC^25^. Moreover, Chen et al. showed that A4.1 at concentrations of 25 µg/ml, 50 µg/ml and 100 µg/ml specifically inhibited TSP1-induced stimulation of G_1_/S cyclin-dependent kinase 2 and entry into S-phase of RASMC. Moreover, high concentrations of A4.1 inhibited FBS-stimulated [^3^H]thymidine incorporation in embryonic fibroblasts isolated from wild type but not p21-deficient mice^28^.

### PDGF-BB induced proliferation was partially inhibited by MiR-21-3p inhibitors and MiR-21-5p inhibitors in ACA SMC but not ITA SMC

MicroRNAs (MiRNAs) are noncoding single-stranded RNAs that regulate post-transcriptional control of gene expression. Formation of the mature form of MiR-21 is a two-step process including cleavage of the stem loops of the primary transcripts of MiR-21 into a ~ 70 nt hairpin precursor, labeled as pre-MiR-21^29^. In SMC, MiR-21 has been widely studied and implicated in several growth responses, including TSP1-induced migration, proliferation, and ERK 1/2 phosphorylation in human aortic SMC^30^. Both ACA SMC and ITA SMC expressed MiR-21 and pre-MiR-21 (Fig. 3C). Expression of MiR-21 and pre-MiR-21 was numerically higher in ACA SMC compared to ITA SMC but the differences did not reach statistical significance.

Consistent with the results from the anti-TSP1 blocking antibody studies, we found that pretreatment with microRNA-21 inhibitors miR-21-3p inhibitors (50 nM) or miR-21-5p inhibitors (50 nM) blocked PDGF-BB induced proliferation of ACA SMC but not ITA SMC (Fig. 3D and E). These results were consistent in the ACA SMC lines from 3 distinct donors and in the ITA SMC lines from 3 distinct donors. In ACA SMC, MiR-21-3p inhibitors and MiR-21-5p inhibitors reduced PDGF-BB-induced proliferation by 49 ± 5% and 50 ± 5%, respectively, when data from all 3 cell lines were combined.

### TSP1 stimulates proliferation of ACA SMC and ITA SMC

**Figure 4:**
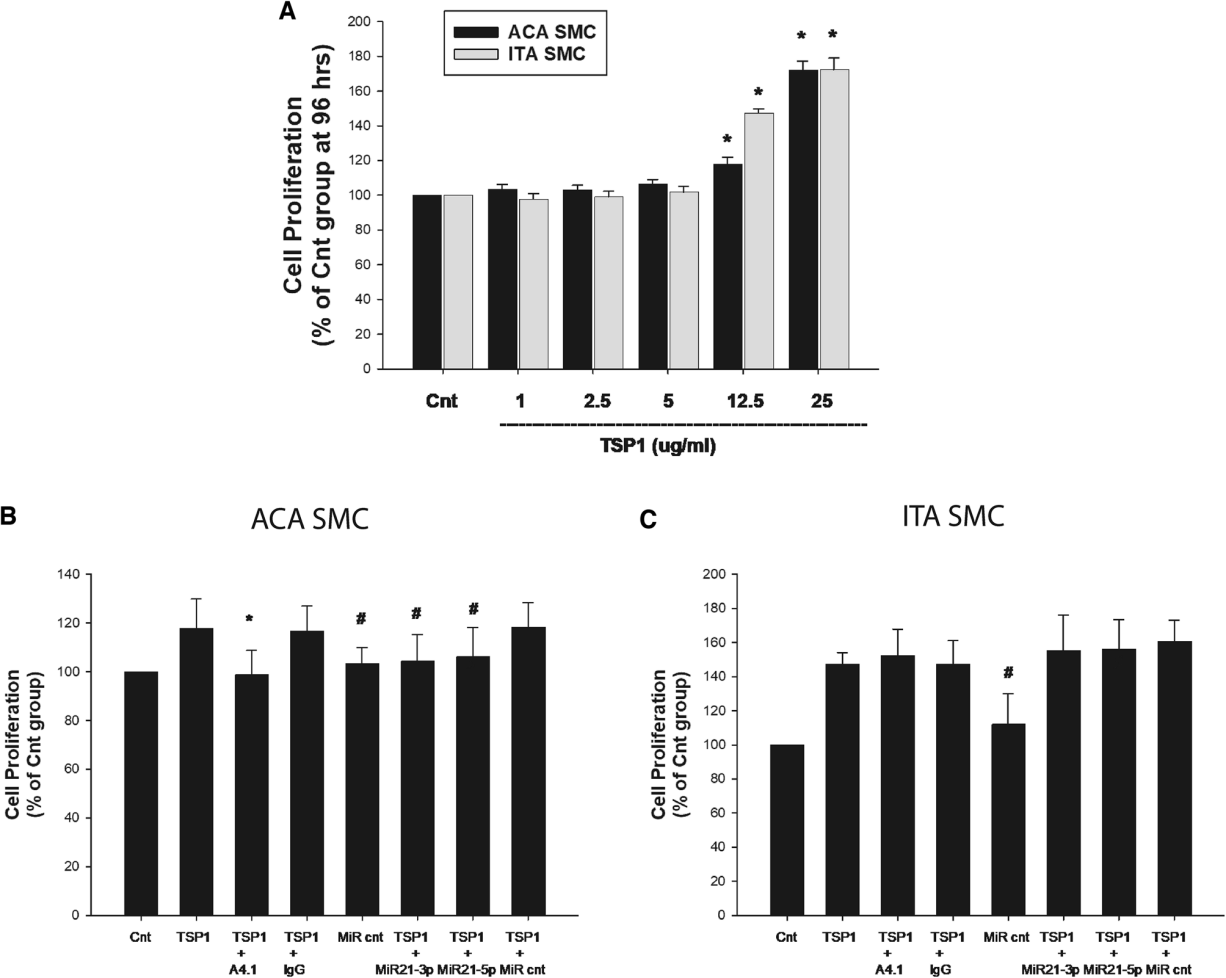
The effects of inhibitors on exogenous TSP1-induced proliferation of ACA SMC and ITA SMC. Human SMC derived from 3 distinct ACA donors and 3 distinct ITA donors were growth arrested and were treated with either vehicle (Cnt) or TSP1 (various concentrations). The results from the different ACA cell lines and different ITA cell lines were consistent and thus the data is presented as an aggregate of the ACA SMC and ITA SMC lines (**A**). In panels (**B**) and (**C**), growth arrested SMC were pretreated with vehicle, anti-TSP-1 antibody A4.1 (10 µg/ml), a non-specific IgG (10 µg/ml), 50 nM miR21-3p inhibitor, 50 nM miR21-5p inhibitor or 50 nM MiRCnt for 30 min prior to treatment with vehicle or TSP1 (12.5 µg/ml). [*—*p* < 0.05 compared to TSP1 + IgG and #—*p* < 0.05 compared to TSP1 + MiR cnt].

To determine whether there were differential growth responses of ACA SMC and ITA SMC to exogenous TSP1, dose–response curves were performed. Results showed that 1 µg/ml, 2.5 µg/ml or 5 µg/ml of TSP1 had no effect on proliferation of ACA SMC or ITA SMC, whereas 12.5 µg/ml and 25 µg/ml of TSP1 stimulated proliferation of ACA SMC and ITA SMC (Fig. 4A).

A4.1, at a concentration of 10 µg/ml, completely inhibited proliferative responses to TSP1 in ACA SMC (Fig. 4B). Similarly, MiR-21-3p inhibitors and MiR-21-5p inhibitors reduced TSP1-induced proliferation in ACA SMC (Fig. 4B). In contrast, neither A4.1, nor the microRNA-21 inhibitors had any effect on TSP1-induced proliferation of ITA SMC (Fig. 4C).

### PDGF-BB stimulates production of TSP1 in ACA SMC but not ITA SMC

**Figure 5:**
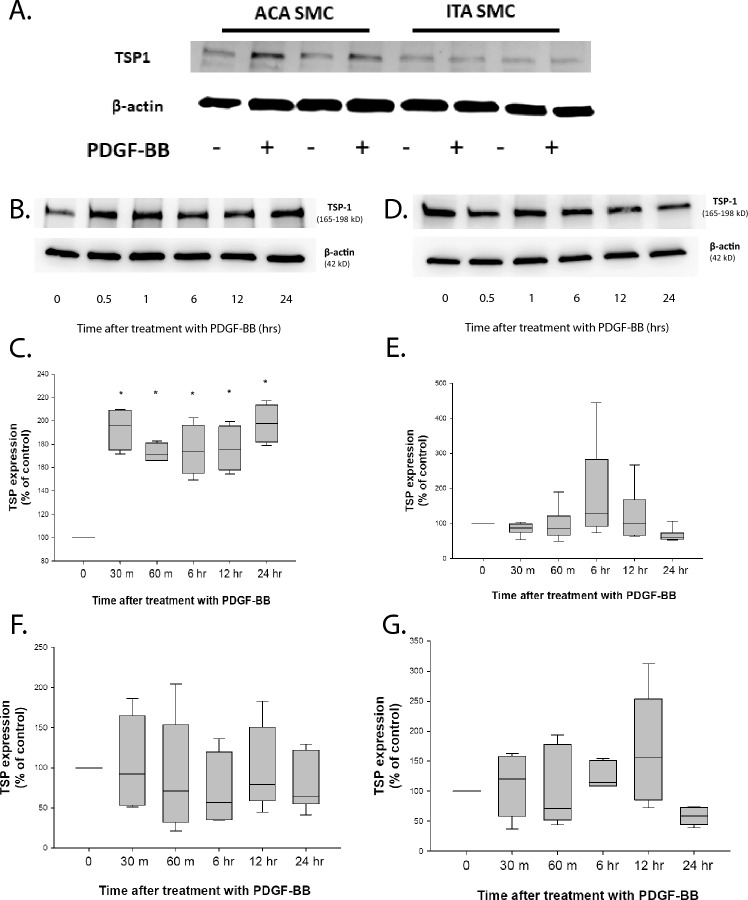
Expression and secretion of TSP1 in PDGF-BB-treated ACA SMC and ITA SMC. Human SMC derived from 3 distinct ACA donors (**A**) and 3 distinct ITA donors (**B**) were growth arrested as described in Methods and then treated with either vehicle or PDGF-BB (20 ng/ml) for various time periods. TSP1 expression and β-actin expression were determined as described in Methods. Panel A shows a representative blot of lysate from ACA SMC treated with PDGF-BB for 24 h and lysate from ITA SMC treated with PDGF-BB for 24 h run on the same gel. A time course of TSP1 expression is shown in panels B–E. SMC isolated from ACA (**B** and **C**) and SMC isolated from ITA (**D** and **E**) were treated with PDGF-BB (20 ng/ml) for various times and representative blots are shown in B and D with results of 6 independent experiments shown in C and E. [*—*p* < 0.05 compared to basal levels]. In panels F and G, SMC were treated with PDGF-BB (20 ng/ml) for the indicated time points, and then conditioned medium was harvested and concentrated. This was followed by Western blot analysis with anti-TSP1 antibody as described in Methods. There were no statistically significant differences, compared to t = 0, for any of the time points in F or G.

We next sought to evaluate TSP1 production by ACA SMC and ITA SMC following treatment with PDGF-BB. Basal TSP1 levels were similar in ACA SMC and ITA SMC when normalized for beta actin production (Fig. 5A). TSP1 levels in lysate from PDGF-BB treated ACA SMC increased beginning 30 min after treatment and remained elevated for at least 24 h (Fig. 5B and C). In contrast, there was no increase in TSP1 levels in PDGF-BB treated ITA SMC at any time point up to and including 24 h (Fig. 5D and E).

Secretion of TSP1 by ACA SMC and ITA SMC was estimated by measuring TSP1 levels in cell supernatant under basal conditions and following treatment with PDGF-BB. Results showed that treatment of ACA SMC and ITA SMC with PDGF-BB for varying amounts of time between 30 min and 24 h was not associated with any measurable increase in the levels of TSP1 in conditioned media in either cell type (Fig. 5F and G). No increase was seen when comparing samples containing an equal amount of protein or samples with equal volumes of conditioned media (data not shown).

## Discussion

The resistance of the ITA to the development of atherosclerosis compared to coronary arteries is largely unexplained and has important clinical implications. ITAs are commonly used during coronary artery bypass grafting and rarely have atherosclerotic changes even in patients with severe, multi-vessel coronary artery disease. A paired postmortem study found that 71% of saphenous vein grafts but only 6% of ITA grafts had histologically proven atherosclerotic changes after a mean follow-up of 56 months in a group of patients with severe coronary artery disease^31^. Patency rates at 10 years are as high as 96%^32^and the presence of an ITA graft is an independent predictor of survival in patients undergoing coronary artery bypass grafting^33^.

The current study provides evidence of important differences in ITA SMC vs ACA SMC in morphology and proliferative responses to TSP1, PDGF-BB and FBS. SMC from ITA were larger, slower growing and had a less robust response to FBS than did SMC from ACA. When grown to confluence, ACA SMC had a classic SMC “hill-and-valley” pattern whereas ITA tended to grow as a monolayer and had a more spindled appearance. The concept that SMC are heterogeneous is widely accepted, and distinct “spindle” and “rhomboid” phenotypes have been identified with rhomboid cells being more proliferative and migratory than spindle cells and the more prevalent phenotype in vascular neointimal lesions^34^. Interestingly, distinct phenotypes of SMC have been isolated from the media of human ITA with the predominant type being ‘epithelioid’ clones that were polygonal at confluence, expressed smooth muscle markers including α-actin and SM-myosin heavy chains and grew faster in response to serum than did ‘spindle-shaped clones^35^.’

A novel finding of the present study is that TSP1- and PDGF-BB-induced proliferation were inhibited by anti-TSP1 antibody A4.1 and MiR-21 inhibitors in ACA SMC but not in ITA SMC. These findings were consistent in ACA primary SMC lines isolated from 3 different donors and ITA primary SMC lines isolated from 3 different donors. TSP1 exists as a homotrimeric glycoprotein with each monomer containing an N-terminal globular module, a central stalk region, and a globular C-terminal assemblage. A4.1 recognizes an epitope in the N-terminal half of the central stalk-like region of TSP1 which may not be important in TSP1-induced effects in ITA SMC. More studies are needed to explore this finding but the actions of the different domains of TSP1 have been shown to vary by cell type and species as a function of extracellular matrix content and cell surface receptors^36^.

These results suggest that there are important differences in the function of, and responses to, TSP1 in ACA SMC vs ITA SMC and add to the existing evidence that different SMC lines have differential mitogenic responses to TSP1. Isenberg et al.^37^ reported that SMCs from TSP1 null mice were selectively deficient in proliferative responses to PDGF-BB but not FBS whereas early studies utilizing RASMC reported that autocrine TSP1 was functionally essential for proliferative responses to FBS^26^. We have previously reported that 7E3, a monoclonal anti- α_v_β_3_ integrin antibody and eptifibatide, a small-molecule inhibitor of α_v_β_3_ blocked proliferative responses to exogenous TSP1 but not to PDGF-BB suggesting that TSP1 was not necessary for proliferative responses to PDGF-BB in the human aortic SMC used in that study^15,38^.

TSP1 levels as determined by Western blotting increased in response to treatment with PDGF-BB in ACA SMC but not ITA SMC. Moreover, while TSP1 was essential for maximal proliferative responses to PDGF-BB in ACA SMC, our data suggests that it does not need to be secreted in order to mediate this effect. While increases in TSP1 levels in cell lysate in ACA SMC were apparent within 30 min of treatment with PDGF-BB with elevated levels present for at least 24 h, we detected no increase in TSP1concentrations in conditioned media in PDGF-BB treated ACA SMC at multiple times points up to 24 h. These results are consistent with those reported by Isenberg et al.^37^ who found that effects of TSP1 on proliferative responses of mouse SMC to PDGF-BB were not dependent upon TSP1 secretion.

A novel finding of the current study is that MiR-21 inhibitors reduced proliferative responses to TSP1 and PDGF-BB in ACA SMC but not ITA SMC. MiR-21 inhibitors can reduce de novo synthesis and/or cause rapid degradation of MiR-21, suggesting that sufficient levels of MiR-21 are important in TSP1- or PDGF-BB-treated ACA SMC but not necessarily ITA SMC. Our results are similar to those observed by Zheng et al.^39^ when comparing SMC isolated from aorta of Wistar-Kyoto rats (WKR) to SMC isolated from aorta of spontaneously hypertensive rats (SHR). MiR-21-3p levels were approximately 50% higher in SMC from SHR and inhibition of miR-21-3p attenuated proliferation, migration and phenotypic transformation of SMC from SHR but not SMC from WKR. Interestingly, overexpression of miR-21-3p promoted proliferation, migration and phenotypic transformation of SMC from both WKR and SHR.

There is accumulating evidence that MiR-21 regulates vascular SMC cell responses to injury. MiR-21 was the most up-regulated microRNA in human atherosclerotic arteries compared with normal arteries^40^ and inhibition of MiR-21 blocked neointimal formation both in a humanized animal model in which balloon-injured human ITA were transplanted into Rowett nude rats^41^ and in a rat carotid artery injury model^42^. Previous studies of cultured SMC have shown that inhibition of MiR-21 reduced serum-induced proliferation of RASMC^42^, PDGF-BB − induced migration and proliferation of human coronary artery SMC^40^ and TSP1-induced proliferation and migration of human aortic SMC^30^.

There are several limitations to the current study. First, the studies were performed in cell culture and the phenotype of SMC in culture vary with cell density, growth state, presence or absence of serum, and many other known and unknown variables. While SMC grown in culture can assume non-physiologic phenotypes, the consistency of the findings from ACA SMC lines isolated from 3 distinct donors and ITA SMC lines isolated from 3 distinct donors adds support to the hypothesis of significant differences between these types of SMC. Second, TSP1 preparations from platelets can be contaminated by TGFβ1 but it is unlikely that the proliferative effects observed with high concentrations of platelet-derived TSP1 were due to contamination by TGFβ1 as recombinant TGFβ1, even at higher concentrations, had no effect on proliferation of either SMC type (data not shown). Lastly, the ACA SMC lines and ITA SMC lines were not derived from the same donor. This is unlikely to have affected the results given the consistent results across cell lines.

In summary, the results of this study show that SMC from ITA and SMC from ACA, when grown in culture, have important differences in morphology, proliferative capacity and in responses to TSP1 and PDGF-BB. These differences were consistent across ACA SMC isolated from 3 distinct donors and ITA SMC lines isolated from 3 distinct donors. Further studies are needed comparing ACA SMC and ITA SMC with the goal of identifying atheroprotective mechanisms in ITA SMC.

### Supplementary Information

Supplementary Information.

## Data Availability

The datasets used and/or analyzed during the current study are available from the corresponding author on reasonable request.
