# Supplementary material for: Differential responses to thrombospondin-1 and PDGF-BB in smooth muscle cells from atherosclerotic coronary arteries and internal thoracic arteries
Source: Sci Rep. 2024 Jul 9;14:15847. doi: 10.1038/s41598-024-66860-x (PMC11233497; doi:10.1038/s41598-024-66860-x)
Supplement: Supplementary file 1 — Supplementary Information. [file 41598_2024_66860_MOESM1_ESM.pdf]

Ref: Submission ID d6f59930-5311-4d50-b775-ba81a5888572

Differential responses to thrombospondin and PDGF-BB in smooth muscle cells from atherosclerotic coronary arteries and internal thoracic arteries.

Pathak and Stouffer

Figure 1C

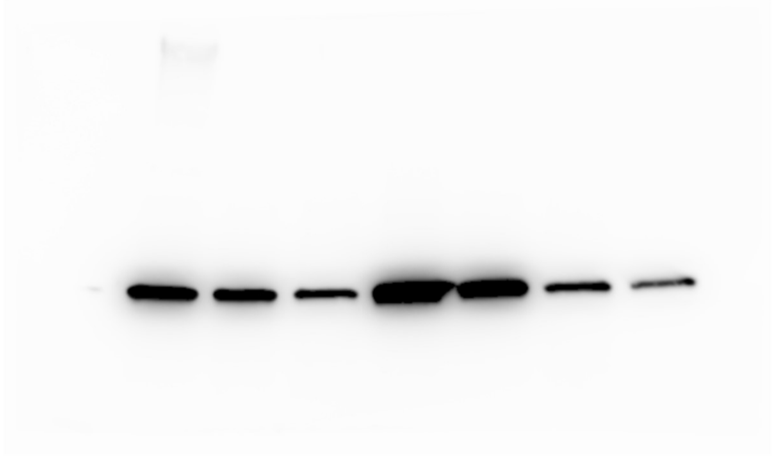

Alpha-actin (43 kD) blot

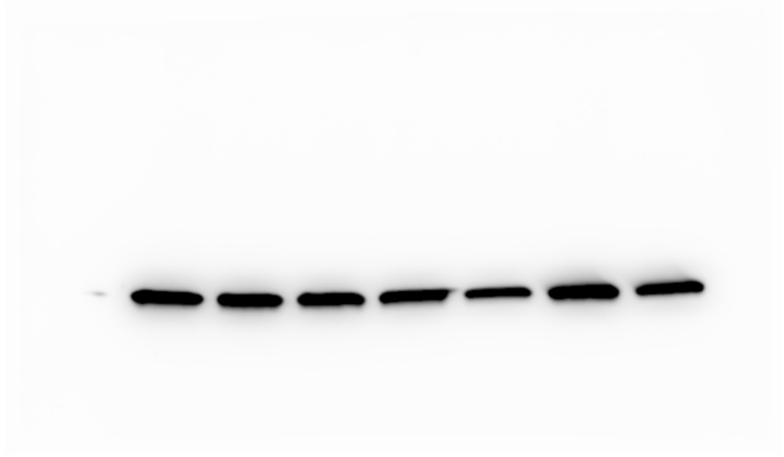

β-actin (42 kD) blot

Figure 1D

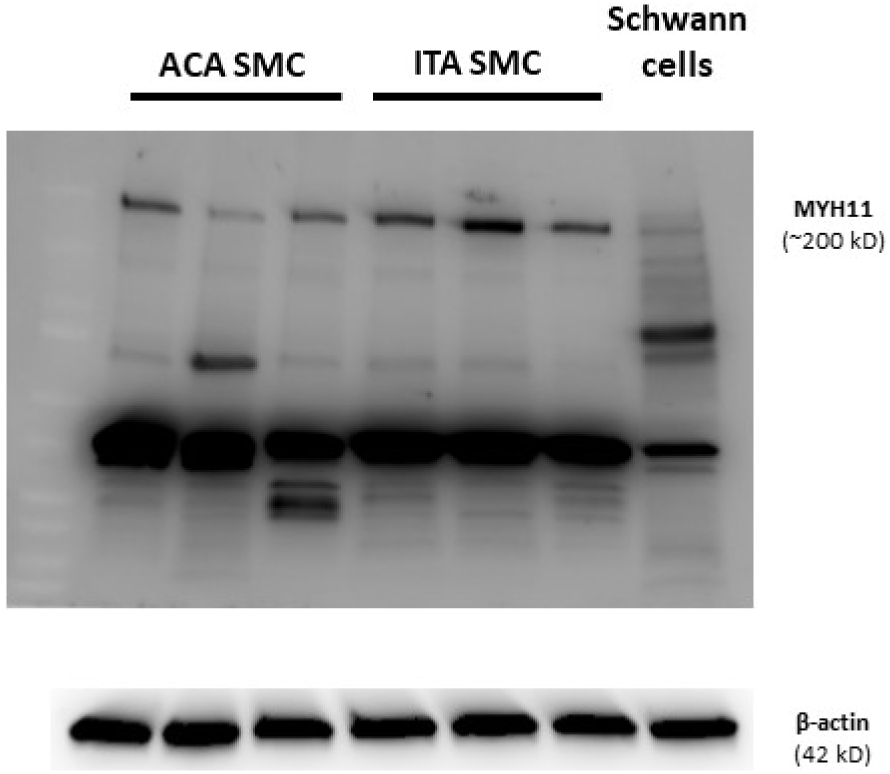

Figure 1E

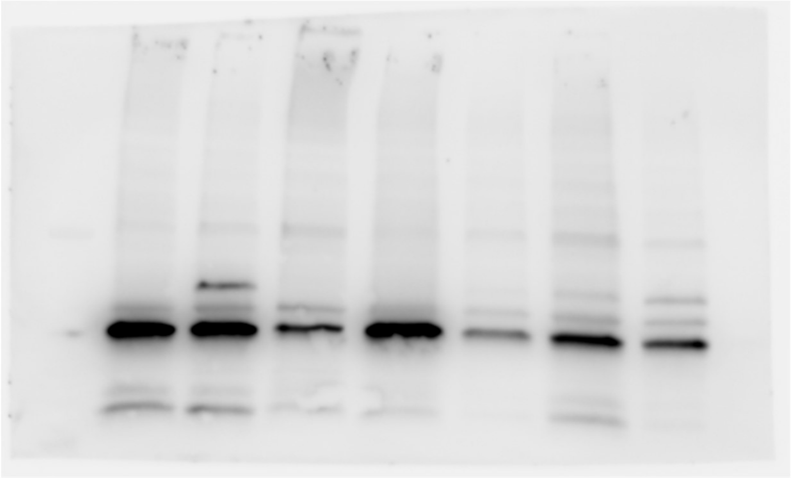

Calponin (33-36 kD)

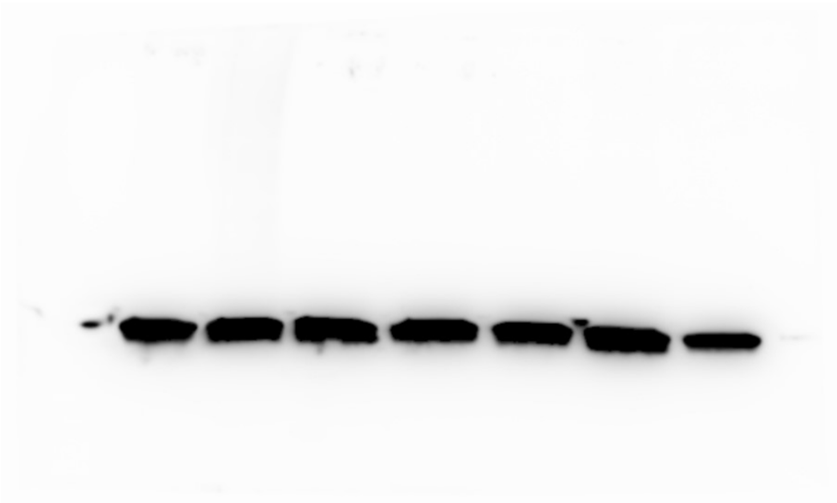

β-actin (42 kD) blot

Figure 1F

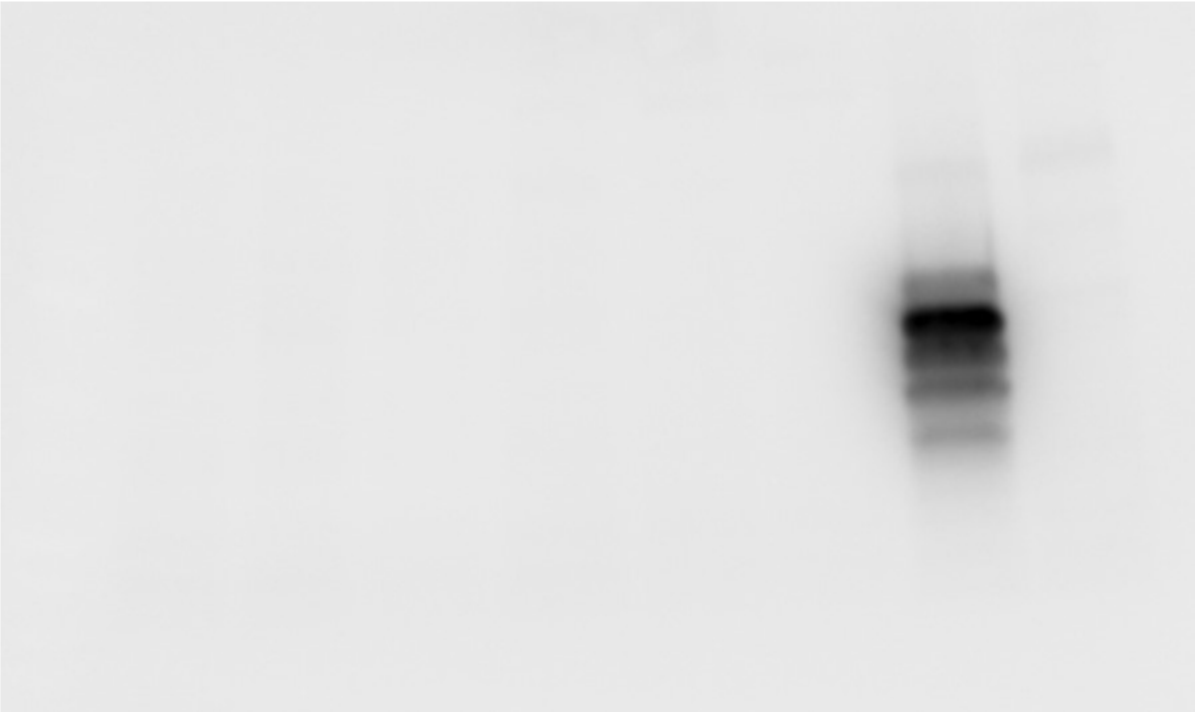

Sox-10 (62 Kd)

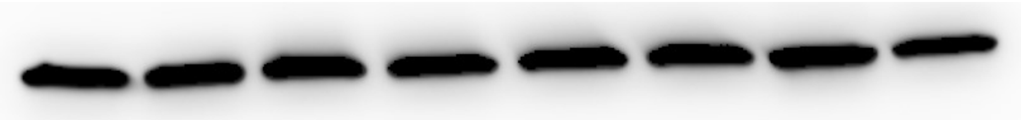

β-actin (42 kD) blot

Figure 1G

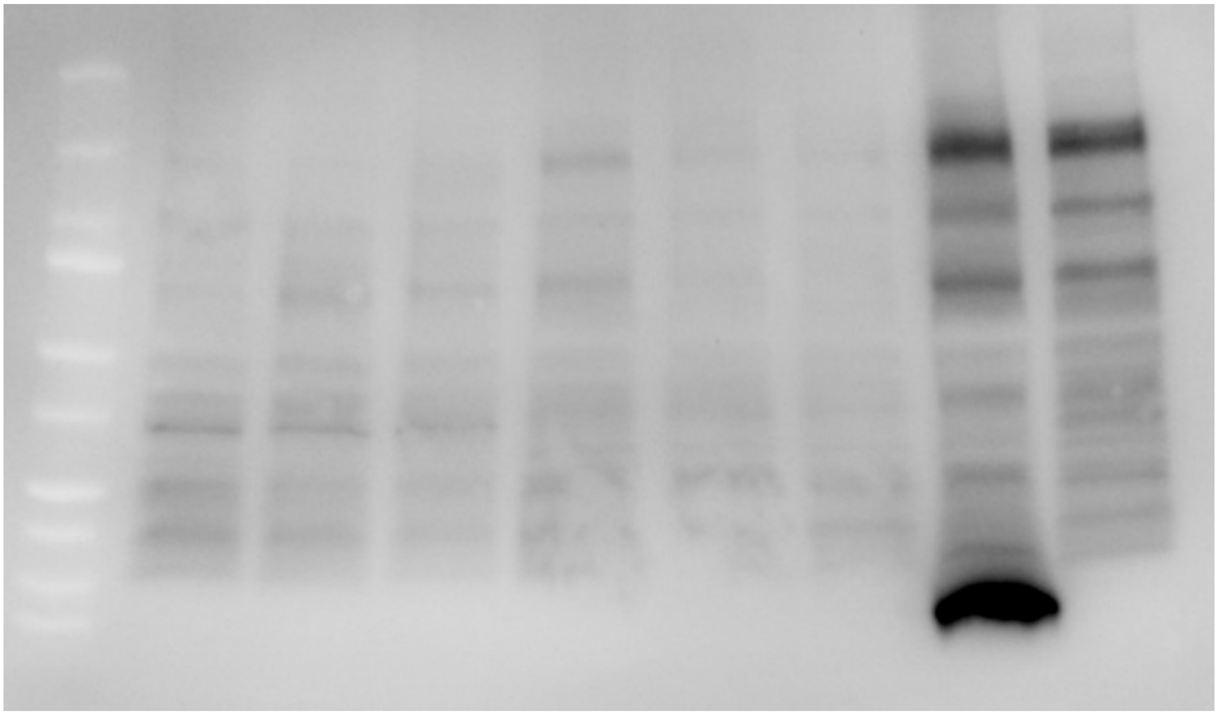

S100B (10 Kd)

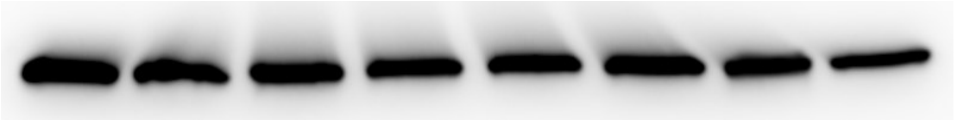

β-actin (42 kD) blot

Figure 5A

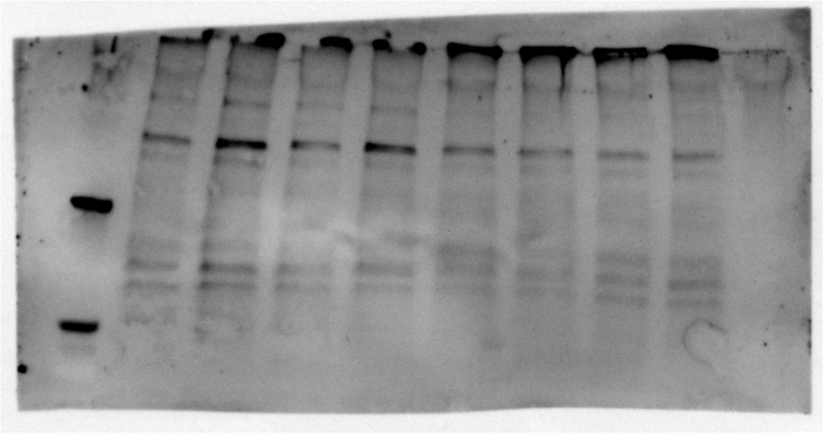

TSP1 (165 – 198 kD) blot

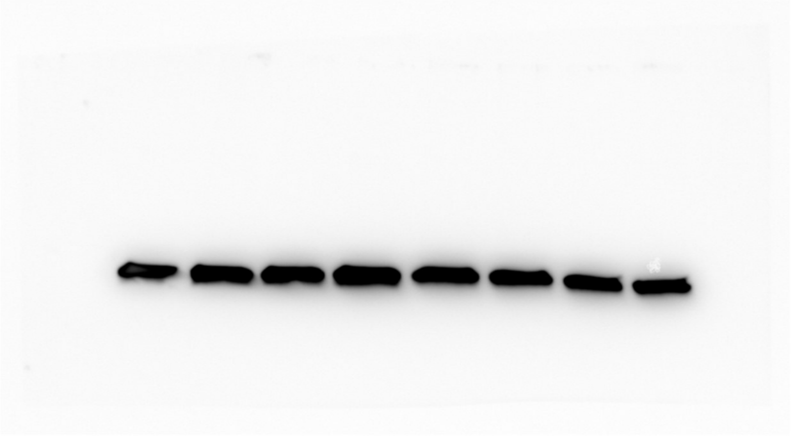

β-actin (42 kD) blot

Figure 5B

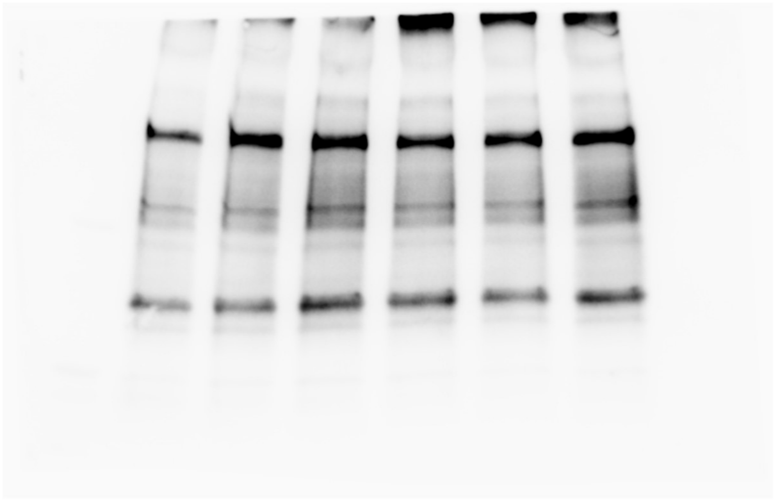

TSP1 (165 – 198 kD) blot

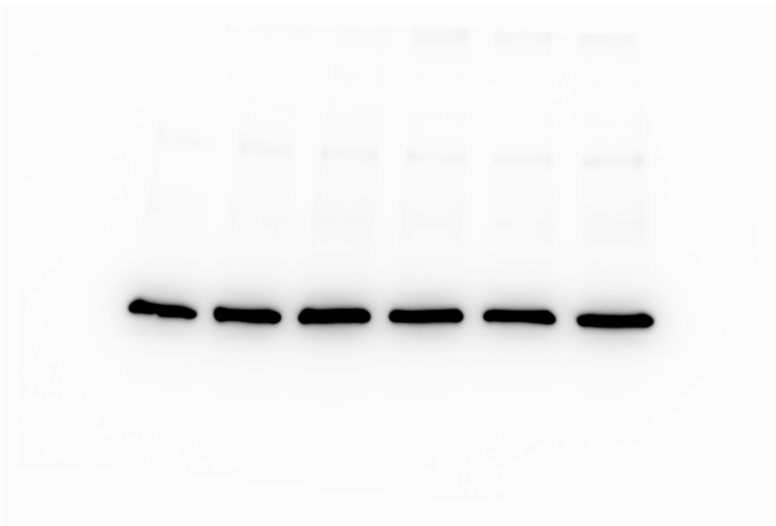

β-actin (42 kD) blot

Figure 5D

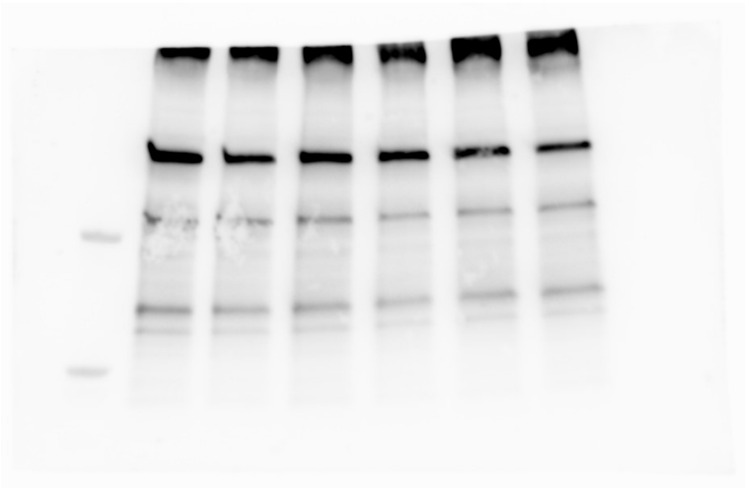

TSP1 (165 – 198 kD) blot

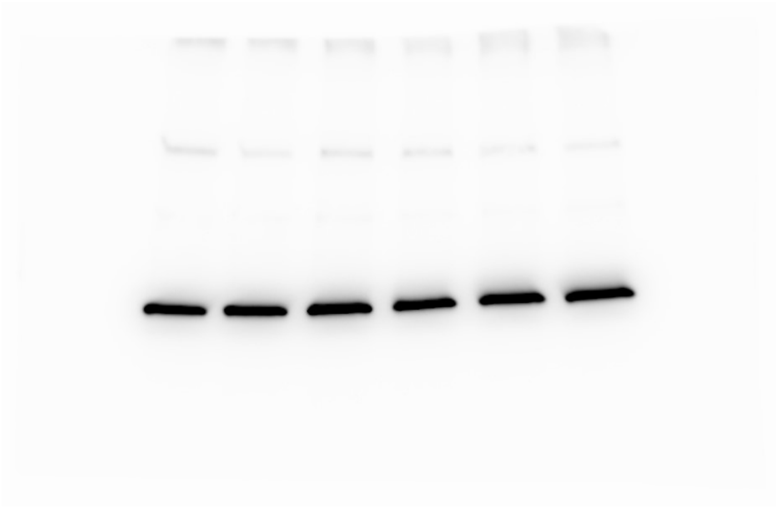

β-actin (42 kD) blot
